# A comparative analysis of risk stratification tools for emergency department patients with chest pain

**DOI:** 10.1186/1865-1380-7-10

**Published:** 2014-02-07

**Authors:** Ellen Burkett, Thomas Marwick, Ogilvie Thom, Anne-Maree Kelly

**Affiliations:** 1 Emergency Department, Princess Alexandra Hospital, Brisbane, Australia; 2 Menzies Research Institute, University of Tasmania, Hobart, Australia; 3 Emergency Department, Nambour General Hospital, Nambour, Australia; 4 School of Public Health, Faculty of Health, Queensland University of Technology, Brisbane, Australia

**Keywords:** Chest pain, Emergency department, Risk score, TIMI, Goldman risk score

## Abstract

**Background:**

Appropriate disposition of emergency department (ED) patients with chest pain is dependent on clinical evaluation of risk. A number of chest pain risk stratification tools have been proposed. The aim of this study was to compare the predictive performance for major adverse cardiac events (MACE) using risk assessment tools from the National Heart Foundation of Australia (HFA), the Goldman risk score and the Thrombolysis in Myocardial Infarction risk score (TIMI RS).

**Methods:**

This prospective observational study evaluated ED patients aged ≥30 years with non-traumatic chest pain for which no definitive non-ischemic cause was found. Data collected included demographic and clinical information, investigation findings and occurrence of MACE by 30 days. The outcome of interest was the comparative predictive performance of the risk tools for MACE at 30 days, as analyzed by receiver operator curves (ROC).

**Results:**

Two hundred eighty-one patients were studied; the rate of MACE was 14.1%. Area under the curve (AUC) of the HFA, TIMI RS and Goldman tools for the endpoint of MACE was 0.54, 0.71 and 0.67, respectively, with the difference between the tools in predictive ability for MACE being highly significant [chi^2^ (3) = 67.21, *N* = 276, *p* < 0.0001].

**Conclusion:**

The TIMI RS and Goldman tools performed better than the HFA in this undifferentiated ED chest pain population, but selection of cutoffs balancing sensitivity and specificity was problematic. There is an urgent need for validated risk stratification tools specific for the ED chest pain population.

## Background

Chest pain remains one of the most common presenting complaints in patients presenting to emergency departments (ED), with >100,000 patients being hospitalized each year in Australia with acute coronary syndromes (ACS) [1]. The morbidity, mortality and economic costs associated with this constitute a significant burden on the Australian health system [2]. In these patients, use of risk stratification tools to predict risk of death, cardiac complications and the pre-test probability of ACS has been demonstrated to aid clinicians in appropriate prioritizing of patients for investigations [3,4] and assists in identification of those at higher risk who might benefit most from potent drug therapies or an early invasive therapeutic approach [5-10]. Risk stratification tools have further been shown to allow patients to be better informed of their prognosis [7], improve cost-effectiveness while minimizing unnecessary treatment complications [11] and reduce unnecessary admissions to inpatient monitored beds, without increasing complications, thereby potentially having a positive impact on access block [3,6,12,13].

Recently, in Queensland, a suite of clinical pathways for the management of patients presenting to public hospitals with chest pain was developed [14], with application incentivized by practice improvement payments [15]. These pathways rely on risk stratification utilizing the National Heart Foundation of Australia risk stratification tool (HFA), perhaps the most prominent risk stratification utilized in Australia [16]. Although the individual components of this tool are evidence based and the tool was developed by consensus of an expert panel, it was designed for risk stratification of patients with ACS rather than for the undifferentiated ED chest pain population. There are conflicting data regarding its performance in ED chest pain populations [17,18].

The aim of this prospective observational study was to compare the performance of three methods of risk stratification, namely, the National Heart Foundation of Australia risk stratification tool (HFA) [16], the Goldman score [6] and the Thrombolysis in Myocardial infarction (TIMI) risk score [19] for prediction of a composite outcome of major adverse cardiac events (MACEs) within 30 days of ED attendance.

## Methods

This was a prospective, cohort study undertaken at a single tertiary referral ED. Patients presenting to the ED with non-traumatic chest pain during the preceding 48 h and aged >30 years were eligible for inclusion in the study. Exclusion criteria were the presence of a definitive non-ischemic cause for chest pain, isolated angina-equivalent symptoms, trauma-related chest pain, cardiac arrest on arrival to the ED, patients with ECG criteria for ST-elevation myocardial infarction (MI) on arrival to the ED and inability to provide informed consent.

Data collection occurred during the ED presentation on weekdays that a trained research nurse was available. Follow-up was undertaken by both review of medical records utilizing a standardized data collection tool and a phone call to the patient (or proxy if the patient was not contactable) employing a structured interview at 72 h and 30 days. A single emergency physician, blinded at the time to the patient outcomes, retrospectively undertook the risk stratification process using the prospectively collected data items and initial ED-acquired electrocardiogram.

All patients included in the study had their cardiac risk determined by each of three methods of risk stratification utilizing findings on presentation to the ED (see Table 1). The HFA [16] and Goldman tools classify patients into risk groups with nominal descriptors (e.g., high, low), while the TIMI risk tool derives a score out of seven [9,17].

**Table 1:** Risk stratification tools

**Risk tool**	**Risk category**	**Features**
**HFA**^**16**^	High risk	Presentation with clinical features consistent with ACS and any of:
		• Repetitive or prolonged (>10 min) ongoing chest pain/discomfort
		• Elevation of at least one cardiac biomarker (troponin or CK-MB)
		• Persistent of dynamic ST depression ≥0.5 mm or new T wave inversion ≥2 mm
		• Transient ST segment elevation (≥0.5 mm) in more than two contiguous leads
		• Hemodynamic compromise: systolic BP <90 mmHg, cool peripheries, diaphoresis, Killip class >1 and/or new onset mitral regurgitation
		• Sustained ventricular tachycardia or syncope
		• Left ventricular systolic dysfunction (LVEF <40%)
		• Prior PCI within 6 months or prior CABG
		• Presence of known diabetes or chronic kidney disease (eGFR <60 ml/min) with typical symptoms of ACS
	Intermediate risk	Presentation with clinical features consistent with ACS and any of:
		• Chest pain or discomfort within the past 48 h that occurred at rest or was repetitive or prolonged (but currently resolved)
		• Age >65 years
		• Known coronary artery disease: prior MI with LVEF ≥40% or known coronary lesion >50% stenosis
		• No high-risk ECG changes
		• Two or more of: known hypertension, family history, active smoking and hyperlipidemia
		• Presence of known diabetes or chronic kidney disease (eGFR <60 ml/min) with atypical symptoms of ACS
		• Prior aspirin use
		**AND NOT** meeting the criteria for high-risk NSTEACS
	Low risk	Presentation with clinical features consistent with ACS without intermediate- or high-risk features
		• Onset of angina symptoms within the last month
		• Worsening in severity or frequency of angina
		• Lowering in angina threshold
**TIMI RS**^**19**^	1 point for each positive factor	• Age >65 years
		• Documented prior coronary artery stenosis >50% or prior cardiac catheterization with known disease or PCI or prior CABG or documented prior myocardial infarction
		• 3 or more conventional cardiac risk factors (hypertension, diabetes, cholesterol elevation, family history of coronary artery disease/MI, history of tobacco use)
		• Use of aspirin in the preceding 7 days
		• 2 or more angina events in the past 24 h
		• ST-segment elevation or depression >1 mm
		• Elevated cardiac biomarkers
**Goldman**^**6**^	Very low risk	• No ECG evidence of acute ischemia/MI and none of the following urgent factors:
		▪ Rales above both lung bases
		▪ Systolic BP <100 mmHg
		▪ Unstable IHD (worsening of previously stable angina, new onset of post-infarction angina or angina after a coronary revascularization procedure or pain that was the same as associated with a prior MI)
	Low risk	No ECG evidence of acute ischemia/MI and 1 of above urgent factors
	Moderate risk	No ECG evidence of acute ischemia/MI and 2 or 3 of above urgent factors
		OR ECG evidence of acute ischemia AND 0 or 1 of above urgent factors
	High risk	ECG evidence of AMI alone OR ECG evidence of acute ischemia with 2 or 3 of above urgent factors

*Abbreviations*: ACS acute coronary syndrome, BP blood pressure, CABG coronary artery bypass graft, CK-MB creatine kinase-MB, ECG electrocardiograph, eGFR estimated glomerular filtration rate, LVEF left ventricular ejection fraction, MI myocardial infarction, NSTEACS non-ST elevation acute coronary syndrome, PCI percutaneous coronary intervention.

The primary outcome of interest was MACE within 30 days of ED presentation. MACE components were defined utilizing the American College of Cardiology Clinical Data Standards definitions [20] and included acute myocardial infarction (prevalent and incident), recurrent ischemia requiring urgent revascularization, cardiogenic shock, ventricular arrhythmia requiring emergent intervention or high-grade atrioventricular block requiring treatment, cardiac arrest and all-cause mortality.

For analysis, continuous variables with normal distribution were expressed as medians and interquartile ranges; categorical data were presented as percentages. Group differences in continuous and categorical variables are compared with Kruskal-Wallis and chi-square tests respectively. For each of the risk stratification methods, ROC curves were used to evaluate its predictive performance. Area under the curve (AUC) was utilized as a summary measure for diagnostic accuracy of the prediction tools across the gamut of risk groups [21], with 95% confidence intervals (CI). For the comparison of clinical performance of the risk scores, we chose to include AHA high risk, Goldman high risk and two cutoffs of the TIMI score (≥1 and ≥2). The latter were chosen pragmatically a priori and attempted to balance case discrimination and sensitivity. Inclusion of patients with a TIMI score of zero provides no case discrimination, while using a cutoff of ≥3 has been shown to have a sensitivity <60% [22].

For all comparisons a *p*-value of <0.05 was considered statistically significant. Statistical analysis was performed with Stata, version 10 (College Station, TX, USA). Power calculations were generated as follows: the AUC of the TIMI score has previously been demonstrated to be 0.6 [23], while the Goldman score has been shown to have an AUC of 0.9 [3,6]. The performance of the HFA score was expected to be similar to that of the Goldman. Assuming a correlation between positive and negative groups of 0.4, a sample size of 400 patients was required to distinguish an AUC of 0.85 from one of 0.9, at a *p* value of 0.05 with an 80% power. An interim analysis was performed as approved study duration and funding were nearing their end. The study was terminated early as this interim analysis revealed a clearly statistically significant result. The institutional ethics committee approved the study.

## Results

**Figure 1:**
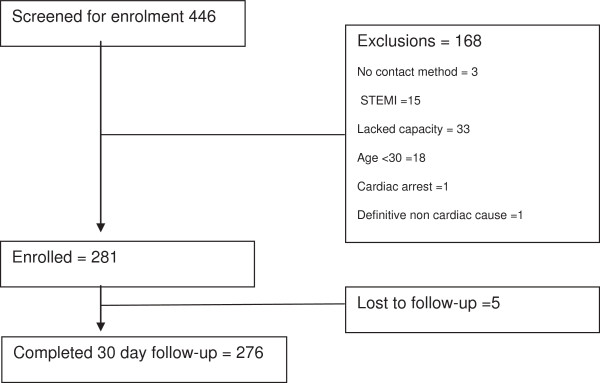
Patient enrollment.

Two hundred eighty-one patients were studied with 276 completing 30-day follow-up. The median age of the study group was 56 years (IQR 47.5-66), with the majority (61.5%) being male (Figure 1). Patient characteristics are summarized in Table 2. Of the 276 patients with 30-day follow-up, 39 (14.1%) had a MACE.

**Table 2:** Patient characteristics

**Feature**		
Age (years; median, interquartile range)		56 (48–66)
Male (%)		61.6
Risk factors (%)	Hypertension	54.1
	Hypercholesterolemia	53
	Smoking history	67.6
	Diabetes mellitus	18.9
	Family history	97
Medications on arrival to hospital (%)	Aspirin within last 7 days	42.7
	Beta-blockers	45.5
	Angiotensin-converting enzyme inhibitors/angiotensin receptor blockers	37
	Statins	52.3
Clinical findings on admission	Heart rate (median, interquartile range)	73 (64–88)
	Systolic blood pressure (median, interquartile range)	140 (126–155)
	Signs of heart failure (%)	8.9
	New ST segment depression or T wave inversion (%)	14.2
	Troponin I > 0.04 ng/ml (%)	11
Results of previous investigations	Left ventricular ejection fraction < 40%	2.8
	Previous coronary artery disease with known > 50% stenosis (%)	20.9
Prior history of revascularization (%)	Percutaneous coronary intervention (%)	16
	Coronary artery bypass graft (%)	10.7

**Figure 2:**
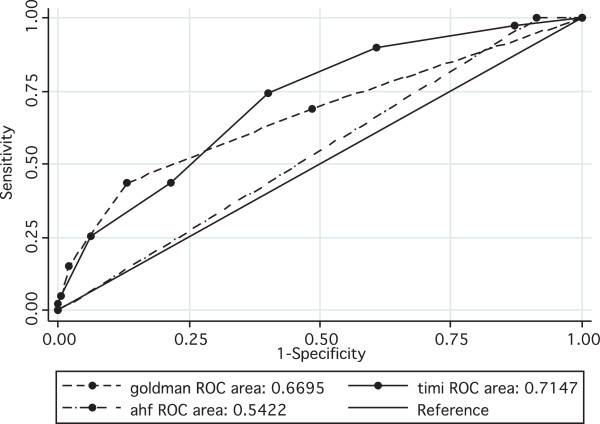
Predictive performance of risk stratification tools.

The predictive performance of the risk stratification tools is shown in Figure 2. AUC for prediction of MACE was poorest for the HFA tool, with an AUC of 0.54 (95% CI 0.45-0.63). The TIMI risk score had the highest AUC of the three tools tested, with an AUC of 0.71 (95% CI 0.63-0.79), while the Goldman tool had an AUC of 0.67 (95% CI 0.57-0.77). The difference between the tools in predictive ability for MACE was highly significant (*p* = 0.0002). There was no statistically significant difference in performance between the Goldman tool and TIMI score. The sensitivity and specificity of the tools are summarized in Table 3.

**Table 3:** Comparative performance of risk stratification tools

**Risk tool**	**Cutoff utilized**	**Sensitivity% (95% confidence interval)**	**Specificity% (95% confidence interval)**	**Likelihood ratio**	**AUC (95% confidence interval)**	***P *** **value**
**HFA**	All high-risk patients	100 (91–100)	8.4 (5.2–12.7)	1.09	0.54 (0.45–0.63)	0.39
**Goldman**	All patients with a risk category of low or higher	69 (52–83)	51 (45–58)	1.43	0.67 (0.57–0.77)	0.0007
**TIMI RS**	TIMI RS of ≥1	97 (87–100)	13 (8.7–18)	1.12	0.71 (0.63–0.79)	<0.0001
	TIMI RS ≥2	90 (76–97)	39 (33–46)	1.48		

Utilizing the chi-square test to compare the AUC for the TIMI, Goldman and HFA tools, the difference between the tools in terms of predictive ability for MACE was found to be highly significant (chi^2^, *N* = 276, *p* <0.0001). Further analysis showed that the HFA tool performance was different from that of each of the other tools, which were similar in performance.

## Discussion

In this study of ED patients with chest pain, where no alternative non-ACS cause was apparent, none of the tools under investigation were ideal. Characteristics of an ideal tool for stratifying risk of chest pain in the ED population would be that it has high sensitivity for risk of MACE, specificity sufficient to enable feasible application and, from a patient perspective, limit exposure to unnecessary investigations or interventions. It would also have data elements that were non-subjective or, at least, had high interobserver reliability. The TIMI risk score had the best performance in stratifying risk for MACE. However, utilizing a cutoff of ≥1 to achieve a sensitivity of 97% resulted in a specificity of only 13%. A higher cutoff of >3 would be required to achieve a more acceptable specificity of 60%; however, the resultant sensitivity of 74% is unacceptable. The sensitivity of the Goldman score (69%) was insufficient to be useful in the ED population, even with the lowest possible cutoff, namely including all patients with a risk higher than ‘very low’ risk. The HFA risk stratification tool had an AUC of 0.54, with 95% confidence intervals (0.45-0.63) disturbingly encompassing an AUC of <0.5. Its sensitivity for MACE, using a cutoff to include all high-risk patients, was 100%, but clearly with a specificity of 8%, its feasibility in ED clinical practice is limited.

The use of an unstructured or individualized approach to ED assessment of chest pain has been shown to be associated with high resource utilization for patients with no coronary artery disease, while concurrently resulting in significant proportions of patients with ACS being missed [24]. Subsequently, emphasis has been placed on utilization of risk stratification tools. Unfortunately, studies employing risk stratification tools for chest pain in the ED setting often have two significant limitations. First, the tools employed have largely been designed for risk stratification of those with known ACS, usually patients admitted to the hospital. Utilizing these tools in the undifferentiated ED patient with chest pain may have a significant impact on both safety and efficiency. Second, many trials of chest pain risk stratification have tested the tools on ED populations where their chest pain is “thought to be of ischaemic origin.” However, as physician discrimination of ACS from other causes of chest pain has previously been shown to be poor, this may result in potentially flawed inferences [24].

The TIMI tool is widely reported as being utilized in the undifferentiated chest pain population in the ED, including as part of rapid diagnostic protocols [25]. In this study, TIMI had comparable sensitivity and specificity for MACE to that found in previously published studies [25], but again highlights that the selection of an appropriate cutoff, balancing sensitivity, specificity and clinical feasibility, is problematic.

Earlier studies identified a higher sensitivity for the Goldman tool in the ED setting than our results [3,26]. This disparity may be accounted for by the fact that these studies differed in their inclusion criteria, with a focus on patients admitted to the hospital, including patients with ST elevation MI, and/or had much earlier follow-up for the endpoint of MACE. Additionally, both the definition of MI and the available cardiac biomarkers have changed considerably since these earlier studies.

The HFA risk stratification tool had an AUC of 0.54. Its high sensitivity for MACE (100%) came at the cost of a specificity of only 8.4%, reflecting classification of 93% of patients as being at “high risk.” This finding is concordant with a previously published study, which similarly questioned the HFA risk stratification tool’s suitability for use in this patient cohort [18]. If the HFA decision support tool [16] were applied to the patient population in this study, it would lead to 93% of ED chest pain patients being admitted to hospital wards and receiving treatments such as heparin. The weight of evidence suggests that the HFA risk stratification tool is inappropriate for use in chest pain pathways in the unselected ED chest pain population.

Other approaches to risk stratification of ED chest pain patients have recently been reported. The HEART score [4,27] was developed in The Netherlands based on clinical experience and literature review rather than database methods. It has five components – history, ECG, age, risk factors and troponin level – which are each rated 0, 1 or 2 based on criteria. In a validation study, it was shown to have better discriminative performance than the GRACE and TIMI risk scores [27]. In multicentre validation, a low HEART score (≤3) had a 1.7% rate of MACE [28]. Of concern is that this score relies in part on subjective assessment of likelihood of ACS for which inter-rater data are scarce.

Another approach was taken by the GRACE investigators with the development of a score aimed at predicting the absence of MACE. The GRACE freedom from events score [29] was developed in an admitted chest pain cohort with likely ACS and has undergone limited external validation on admitted chest pain cohorts [30,31]. No validation in an ED chest pain cohort has yet been published.

A similar approach has been taken in the development of the North American Chest Pain Rule (NACPR) [32]. It aims to identify low-risk ED chest pain patients suitable for early discharge. The rule consists of the absence of five predictors—ischemic ECG changes not known to be old, history of coronary artery disease, pain typical for ACS, initial or 6-h troponin level greater than the 99th percentile and age greater than 50 years. In internal validation, it was 100% sensitive (95% confidence interval 97.2% to 100.0%) and 20.9% specific (95% confidence interval 16.9% to 24.9%) for a cardiac event within 30 days, with 11% of patients being defined as low risk [32]. Its utility has been challenged in an external validation study and comparison to the HEART score [33]. The NACPR identified 4.4% (95% CI 3-6%) for early discharge with 100% (95% CI 98-100%) sensitivity for ACS, while the HEART score identified 20% (95% CI 18-23%) for early discharge with 99% (95% CI 97-100%) sensitivity for ACS. The low proportion of patients identified as low risk in a population with MACE of 22% is a serious threat to this score’s clinical utility. That said, the approach of trying to decide who is safe for discharge rather than identification of high risk is worthy of further exploration.

This study has some limitations that should be considered in interpreting the results. Bias in the verification of events is possible if the inability to contact patients in follow-up coincided with those having events. The potential for verification bias has been minimized by follow-up not only via chart review but also via admissions registry review and with the patient or proxy. While most data were collected prospectively, some were collected retrospectively and so are subject to potential data omission. Some of the data were reliant on patient self-report (e.g., of past history and risk factors). If available in the chart, this was verified; however, otherwise, no attempt was made to verify these data, reflecting the real-world ED clinical interaction. The study was conducted at a single site, and this may have impacted the external validity.

## Conclusions

The TIMI risk stratification score appeared most suitable for use in an undifferentiated ED chest pain population, but selection of an appropriate cutoff is problematic. This study highlights the need for validated risk stratification tools for the ED chest pain cohort that examine not only their safety in terms of their sensitivity, but also their flow and efficiency impacts, as these factors have significant implications for safety for all ED patients.

## Competing interests

Funding was provided for this project by the Queensland Emergency Medicine Research Fund. AMK is co-author of the Australasian guidelines for the management of acute coronary syndromes.

## Authors’ contributions

EB had the concept for the study, all authors had input into study design, EB, TM and OT undertook analysis, all authors had input into interpretation of data, EB and AMK drafted the manuscript with all authors contributing to its refinement and approving the final manuscript.
